# Characterization of Hypoxia-Related Molecular Subtypes in Clear Cell Renal Cell Carcinoma to Aid Immunotherapy and Targeted Therapy *via* Multi-Omics Analysis

**DOI:** 10.3389/fmolb.2021.684050

**Published:** 2021-06-25

**Authors:** Weimin Zhong, Hongbin Zhong, Fengling Zhang, Chaoqun Huang, Yao Lin, Jiyi Huang

**Affiliations:** ^1^ Department of Clinical Medicine, Fujian Medical University, Fuzhou, China; ^2^ Central Laboratory, the Fifth Hospital of Xiamen, Xiamen, China; ^3^ Department of Nephrology, Xiang’an Branch, the First Affiliated Hospital of Xiamen University, Xiamen, China; ^4^ Department of Nephrology, the First Affiliated Hospital of Xiamen University, Xiamen, China; ^5^ Department of Traditional Chinese Medicine, the Fifth Hospital of Xiamen, Xiamen, China; ^6^ Central Laboratory at The Second Affiliated Hospital of Fujian Traditional Chinese Medical University, Collaborative Innovation Center for Rehabilitation Technology, Fujian University of Traditional Chinese Medicine, Fuzhou, China

**Keywords:** clear cell renal cell carcinoma, hypoxia, molecular subtypes, immunotherapy, targeted therapy

## Abstract

**Objective:** Tumor hypoxia is a key factor in resistance to anti-cancer treatment. Herein, this study aimed to characterize hypoxia-related molecular subtypes and assess their correlations with immunotherapy and targeted therapy in clear cell renal cell carcinoma (ccRCC).

**Materials:** We comprehensively analyzed copy number variation (CNV), somatic mutation, transcriptome expression profile and clinical information for ccRCC from TCGA and ICGC databases. Based on 98 prognosis-related hypoxia genes, samples were clustered using unsupervized non-negative matrix factorization (NMF) analysis. We characterized the differences between subtypes concerning prognosis, CNV, somatic mutations, pathways, immune cell infiltrations, stromal/immune scores, tumor purity, immune checkpoint inhibitors (ICI), response to immunotherapy and targeted therapy and CXC chemokines. Based on differentially expressed genes (DEGs) between subtypes, a prognostic signature was built by LASSO Cox regression analysis, followed by construction of a nomogram incorporating the signature and clinical features.

**Results:** Two hypoxia-related molecular subtypes (C1 and C2) were constructed for ccRCC. Differential CNV, somatic mutations and pathways were found between subtypes. C2 exhibited poorer prognosis, higher immune/stromal scores, and lower tumor purity than C1. Furthermore, C2 had more sensitivity to immunotherapy and targeted therapy than C1. The levels of CXCL1/2/3/5/6/8 chemokines in C2 were distinctly higher than in C1. Consistently, DEGs between subtypes were significantly enriched in cytokine-cytokine receptor interaction and immune responses. This subtype-specific signature can independently predict patients’ prognosis. Following verification, the nomogram could be utilized for personalized prediction of the survival probability.

**Conclusion:** Our findings characterized two hypoxia-related molecular subtypes for ccRCC, which can assist in identifying high-risk patients with poor clinical outcomes and patients who can benefit from immunotherapy or targeted therapy.

## Introduction

Renal cell carcinoma (RCC) occupies approximately 2% of all adult cancers globally (Siegel et al., 2019). Clear cell RCC (ccRCC) is the main histological subtype of RCC (∼80%), with extremely poor prognosis (Siegel et al., 2019). For local ccRCC, surgery is the preferred treatment, while immunotherapy, targeted therapy and chemotherapy have been approved for treatment of advanced or metastatic ccRCC (Chen et al., 2019). However, not all patients can respond to above treatments. Recent genomic research has uncovered a distinct complexity of intra- and inter-tumor heterogeneity in ccRCC, which has contribution to the varying prognosis of patients (Gerlinger et al., 2012; Cancer Genome Atlas Research Network, 2013). It is expected to achieve long-term survival of ccRCC patients by improving the ability to identify high-risk patients and further developing personalized treatment based on multi-omics.

Hypoxia is one of the signs of tumor microenvironment. It has been widely regarded as an active participator for ccRCC progression (Jing et al., 2019). Hypoxia-induced changes in gene expression exert critical effects on various cellular and physiological functions, thereby ultimately limiting the prognosis of patients (Vito et al., 2020). The behavior of tumor cells is highly influenced by their surrounding microenvironment. Under hypoxic conditions, tumor cells have remarkably restored their survival and proliferation (Riera-Domingo et al., 2020). For example, the acidic microenvironment induced by hypoxia can promote chemoresistance by inducing epithelial-mesenchymal transition and stem cell-like phenotypes (Damgaci et al., 2018). Especially, hypoxia can drive immune escape in the tumor microenvironment and hinder the success of immunotherapy (Riera-Domingo et al., 2020). Hence, a better understanding of hypoxia-related molecular characteristics may contribute to the progression of cancer immunotherapy research and provide a theoretical basis for clinical trials to help improve treatment effects (Zhang et al., 2020). In this study, we aimed to comprehensively characterize the hypoxia-related molecular subtypes and their clinical implications for immunotherapy and targeted therapy of ccRCC *via* multi-omics data.

## Materials and Methods

### Hypoxia-Related Genes

The “HALLMARK_HYPOXIA” gene sets were downloaded from The Molecular Signatures Database v7.2 (MSigDB; https://www.gsea-msigdb.org/gsea/msigdb) using Gene Set Enrichment Analysis (GSEA) v4.1.0 software (Subramanian et al., 2005), where there were 200 hypoxia genes that were up-regulated in response to hypoxia (Supplementary Table 1).

### Data Collection and Preprocessing

Level 3 RNA sequencing (RNA-seq), somatic mutation data, copy number variation (CNV) data and corresponding clinical information (age, gender, grade, stage, survival status and follow-up information) for ccRCC were retrieved from The Cancer Genome Atlas (TCGA, http://cancergenome.nih.gov/) or the International Cancer Genome Consortium (ICGC, www.icgc.org). Samples with survival time ≥30 days were retained. Consequently, 512 ccRCC samples from TCGA were enrolled as the training set, while 90 samples from ICGC database were included in the external validation set. The two datasets were integrated into the entire set and batch effects were corrected with the “ComBat” algorithm of sva package (Leek et al., 2012).

### Clustering Analysis

Before clustering, univariate cox regression survival analysis was performed to evaluate the correlation between hypoxia genes and overall survival (OS) in TCGA-ccRCC cohort. Consequently, genes with *p* < 0.05 were retained for sample clustering analysis. Then, unsupervized non-negative matrix factorization (NMF) clustering was conducted *via* the NMF package in *R* on the TCGA and ICGC datasets, respectively (Gaujoux and Seoighe, 2010). The *k* value when cophenetic correlation coefficient started to decline was chosen as the optimal number of clusters. Principal components analysis (PCA) and t-distributed stochastic neighbor embedding (t-SNE) were presented to verify the classification performance on the basis of the transcriptome expression profile of above hypoxia-related genes. Kaplan-Meier overall survival (OS) curves were drawn using the survival package in *R*, followed by log-rank test.

### Mutation Estimation

Amplification and deletion variations were evaluated using the Genomic Identification of Significant Targets in Cancer (GISTIC) v2.0 by the genePattern software. Furthermore, somatic mutation data were extracted and the mutation frequencies were counted *via* the MutSigCV algorithm.

### Gene Set Variation Analysis

The GSVA algorithm was used to probe into the distinct signaling pathways between subtypes on the basis of transcriptomic expression profile (Hänzelmann et al., 2013). The gene set of “c2.cp.kegg.v7.1.symbols” was employed as the reference. The enrichment scores of pathways in each sample were calculated and their differences between subtypes were analyzed using the linear models for microarray data (limma) package (Ritchie et al., 2015). Differential pathways were screened with the criteria of false discovery rate (FDR) < 0.05 and |log2 fold change (FC)| >0.2.

### Cell Type Identification by Estimating Relative Subsets of RNA Transcripts

Using the CIBERSORT algorithm, the infiltration levels of 22 kinds of immune cells were estimated for each ccRCC sample in TCGA database. The differences in the immune infiltration levels between subtypes were calculated *via* the Wilcoxon rank-sum test. Infiltrating immune cells were clustered by hierarchical agglomerative clustering based on Euclidean distance and Ward’s linkage.

### Estimation of Stromal and Immune Cells in Malignant Tumors Using Expression Data

The levels of infiltrating stromal and immune cells in ccRCC tissues were estimated for each sample based on the gene expression profiles utilizing the ESTIMATE algorithm (Yoshihara et al., 2013). By combining stromal and immune scores, ESTIMATE scores were determined. Tumor purity of each sample was then calculated according to the ESTIMATE scores.

### Assessment of Immune Checkpoint Inhibitors, Response to Immune Therapy and Tumor Mutation Burden Between Subtypes

The likehood of response to immunotherapy was assessed by the Tumor Immune Dysfunction and Exclusion (TIDE; http://tide.dfci.harvard.edu/login/) website. TMB was defined as the ratio of total count of variants and the whole length of exons. The differences in the expression levels of ICIs, TIDE scores and TMB levels were compared by the Wilcoxon rank-sum test.

### Drug Sensitivity Prediction

The sensitivity of each sample to chemotherapy drugs was predicted by the Genomics of Drug Sensitivity in Cancer (GDSC; https://www.cancerrxgene.org/) database (Yang et al., 2013). The half maximal inhibitory concentration (IC50) was assessed through ride regression utilizing the pRRophetic package in *R*. Furthermore, the predictive accuracy was verified *via* ten-fold cross-verification in the TCGA-ccRCC cohort.

### Differential Expression and Functional Annotation Analysis

Differentially expressed genes (DEGs) were filtered between two molecular subtypes *via* the egdeR package with the cutoff of FDR <0.05 and |log2 FC| ≥2. Their underlying functions were predicted through Gene Ontology (GO) and Kyoto Encyclopedia of Genes and Genomes (KEGG) enrichment analysis *via* the clusterProfiler package in *R* (Yu et al., 2012). The *p*-value was adjusted by Benjamini-Hochberg method. Adjusted *p* < 0.05 was considered significant.

### Screening Small Molecule Drugs

The two gene lists of up- and down-regulated tags were uploaded into the Connectivity map (CMap; http://portals.broadinstitute.org/cmap/) database (Lamb et al., 2006). Candidate small molecular drugs were screened according to the enrichment value and permutation *p*-value. CMap mode-of-action (MoA) analysis was exploited to explore potential mechanisms of action.

### Establishment of a Signature Based on DEGs in Two Molecular Subtypes

Prognosis-related DEGs with *p* < 0.05 were screened by univariate cox regression survival analysis. The least absolute shrinkage and selection operation (LASSO) Cox regression model was constructed *via* the glmnet package (Friedman et al., 2010). ccRCC patients from TCGA database were divided into high- and low-risk groups in line with the cutoff value of risk scores. Kaplan-Meir curves were portrayed to compare the differences in OS and disease-free survival (DFS) between the two groups *via* the survival package. Time-dependent receiver operating characteristic curves (ROCs) for one-, three- and five-years OS and DFS were conducted for assessment of the predictive power of the risk score using the survivalROC package. Multivariate Cox regression analysis was carried out to assess the independency of the risk score for OS and DFS. A forest plot containing the hazard ratio (HR) and 95% confidence interval (CI) of each variable was then drawn *via* survminer package.

### Construction of a Nomogram Model

Clinical factors and the risk score were incorporated into a nomogram for predicting OS and DFS using the rms package in *R*. The scores of variables were given based on their regression coefficients. For each patient, a total score was calculated by adding up the corresponding individual scores of all variables. Then, using conversion function, the probability of outcome of each patient was calculated. The predictive efficacy of the nomogram was investigated by calibration plots.

### Statistical Analysis

All statistical analysis was achieved *via R* language v4.0.2 (https://www.r-project.org/). Comparisons between two groups were presented *via* Wilcoxon rank-sum test. A two-tailed *p*-value <0.05 was considered statistically significant.

## Results

### Characterization of Two Hypoxia-Related Molecular Subtypes with Distinct Clinical Implications for ccRCC

**FIGURE 1:**
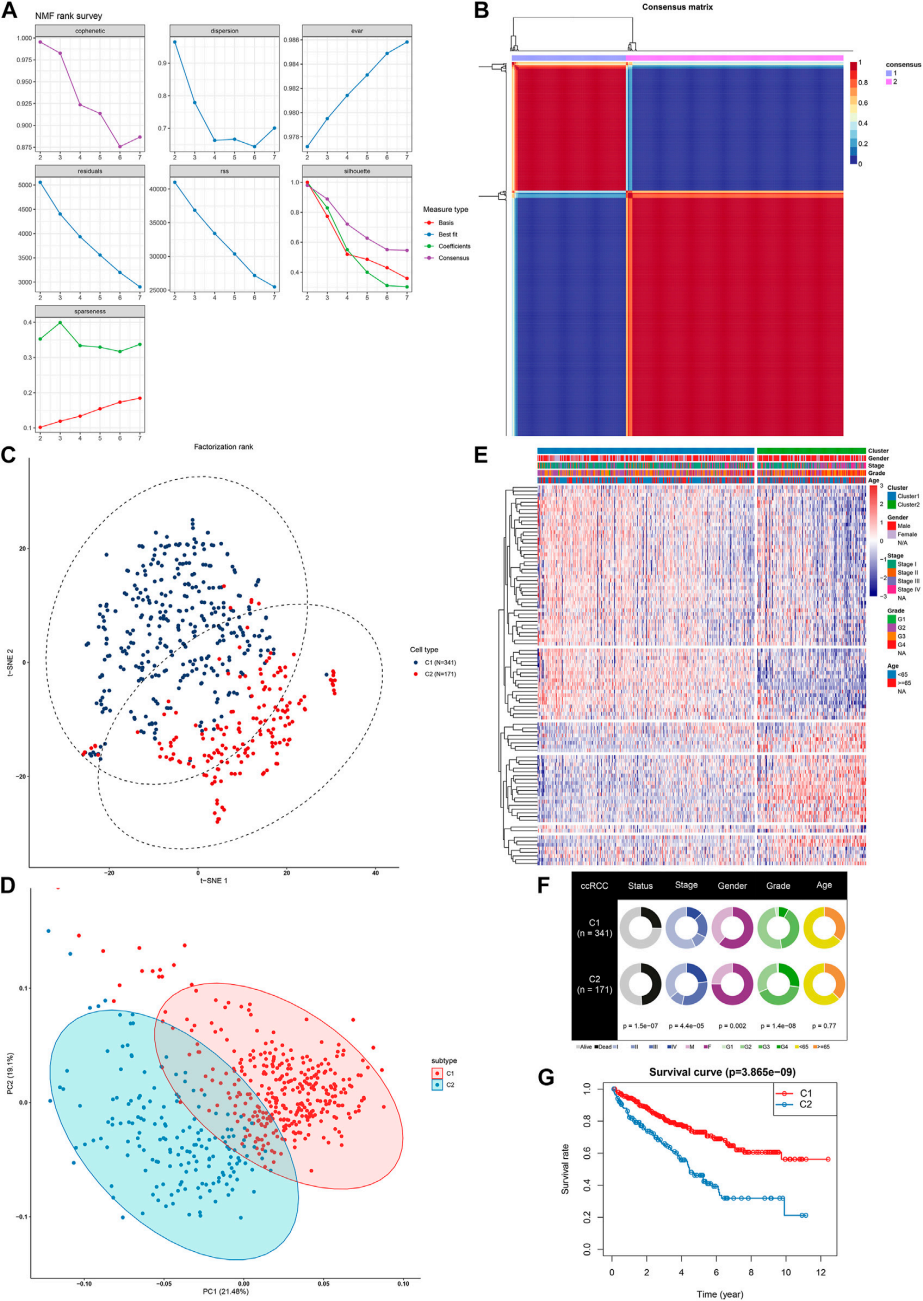
NMF identifies two distinct hypoxia-related molecular subtypes for ccRCC in TCGA-ccRCC dataset **(A)** Factorization rank for k = 2–7 **(B)** The heat map of the consensus matrix when the consensus clustering k = 2. The value range is 0–1. The columns and rows are sorted through hierarchical clustering according to the Euclidean distance of the average link **(C)** The PCA and **(D)** t-SNE scatter plots are in support of the classification into two ccRCC molecular subtypes based on the gene expression profiles. The colors are indicative of samples from two molecular subtypes **(E)** The heatmap visualizing the expression patterns of hypoxia genes in the two subtypes. Samples are clustered according to different clinical features **(F)** Correlation between subtypes and clinical features **(G)** Kaplan-Meier OS curves for the two clusters in TCGA-ccRCC dataset. The assessment of difference was achieved by log-rank test.

200 hypoxia genes were retrieved from the list “HALLMARK_HYPOXIA” gene set. In the TCGA-ccRCC (n = 512) cohort, a total of 98 genes were associated with ccRCC prognosis (all *p* < 0.05), while the other genes could not impact ccRCC prognosis (Supplementary Table 2). Based on the expression profiles of prognostic hypoxia genes, ccRCC samples from TCGA were clustered *via* the NMF package. The optimal value of *k* was determined on the grounds of cophenetic correlation coefficient. When starting from *k* = 2, cophenetic correlation coefficient started to decrease (Figure 1A). The heatmap intuitively showed the consensus matrix when *k* = 2 (Figure 1B). Hence, ccRCC samples were clustered into two molecular subtypes C1 (*n* = 341) and C2 (171). The PCA (Figure 1C) and t-SNE (Figure 1D) supported the classification into two subtypes. As depicted in heatmap, there was a distinct difference in expression patterns of hypoxia genes between subtypes (Figure 1E). Furthermore, we found the significant differences in status (*p* = 1.5e-07), stage (*p* = 4.4e-05), gender (*p* = 0.002) and grade (*p* = 1.4e-08) between subtypes (Figure 1F). The significant prognostic difference was investigated in TCGA-ccRCC cohort, with shorter OS time in C2 than in C1 (*p* = 3.865e-09; Figure 1G). This classification was confirmed in the ICGC dataset (Supplementary Figure 1A–E). However, due to small sample size, the prognosis of patients was not significantly different between subtypes (Supplementary Figure 1F). Therefore, we integrated the samples from the TCGA and ICGC datasets into the entire set after removing batch effects (Supplementary Figure 2A). In the entire set, the two molecular subtypes with distinct prognosis and clinicopathological characteristics were confirmed (Supplementary Figure 2B-F).

### Differential CNV and Somatic Variation Landscape and Subtype-specific Signaling Pathways Between Subtypes

**FIGURE 2:**
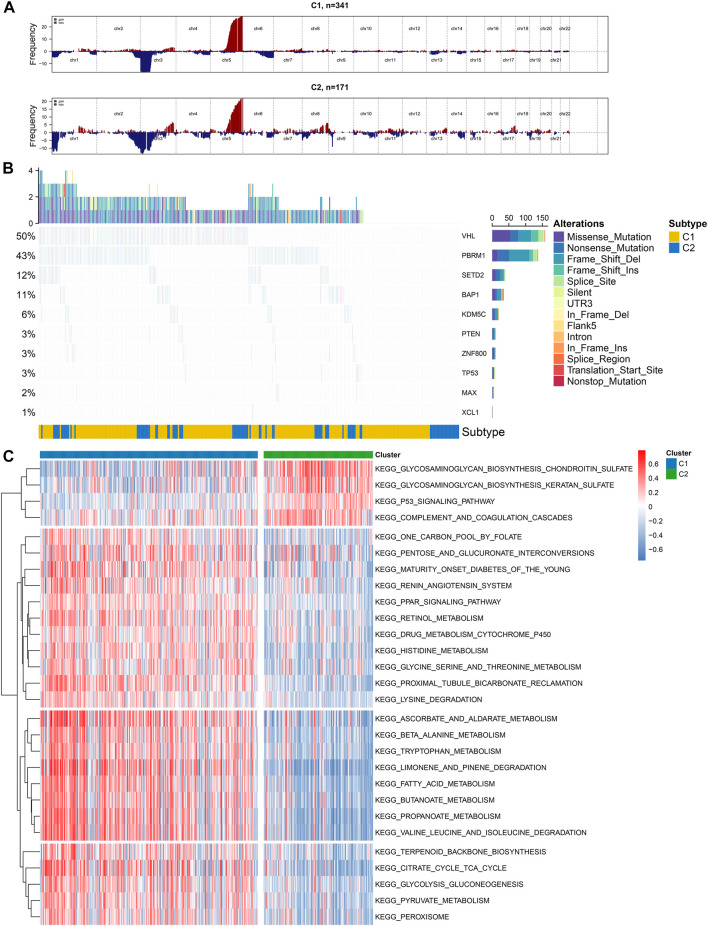
Illustration of copy number and somatic variations and subtype-specific signaling pathways in two hypoxia-related molecular subtypes **(A)** The landscape of copy number alterations in C1 and C2 subtypes **(B)** Oncoplot visualizing the somatic landscape of ccRCC samples in the two subtypes. The top ten genes are ranked on the grounds of the mutation frequency. Different mutation types are annotated by different colors on the right **(C)** Heatmap showing subtype-specific signaling pathways.

We visualized the mutation frequencies of CNV in ccRCC samples from two subtypes (Figure 2A). The chromosome 5 occurred the most frequent amplification both in the C1 and C2 subtypes. Meanwhile, the chromosome 2 and 3 harbored the most frequent deletion sites. The frequency of amplification and deletion in C2 was more common than that in C1. The MutSigCV algorithm was applied to compare the frequency of somatic mutation between C1 and C2. Genetic alterations of ccRCC mainly consist of those that control cellular oxygen induction (such as VHL) as well as maintaining chromatin states (such as PBRM1). Consistently, among ccRCC samples, VHL exhibited the most frequent mutations (50%), followed by PBRM1 (43%) and SETD2 (12%; Figure 2B). We further probed into subtype-specific signaling pathways by GSVA (Supplementary Table 3). As depicted in Figure 2C, p53 signaling pathway was down-regulated C1 compared to C2, and metabolism-related pathways were up-regulated in C1 than C2.

### More Sensitivity to Immunotherapy for Molecular Subtype 2

**FIGURE 3:**
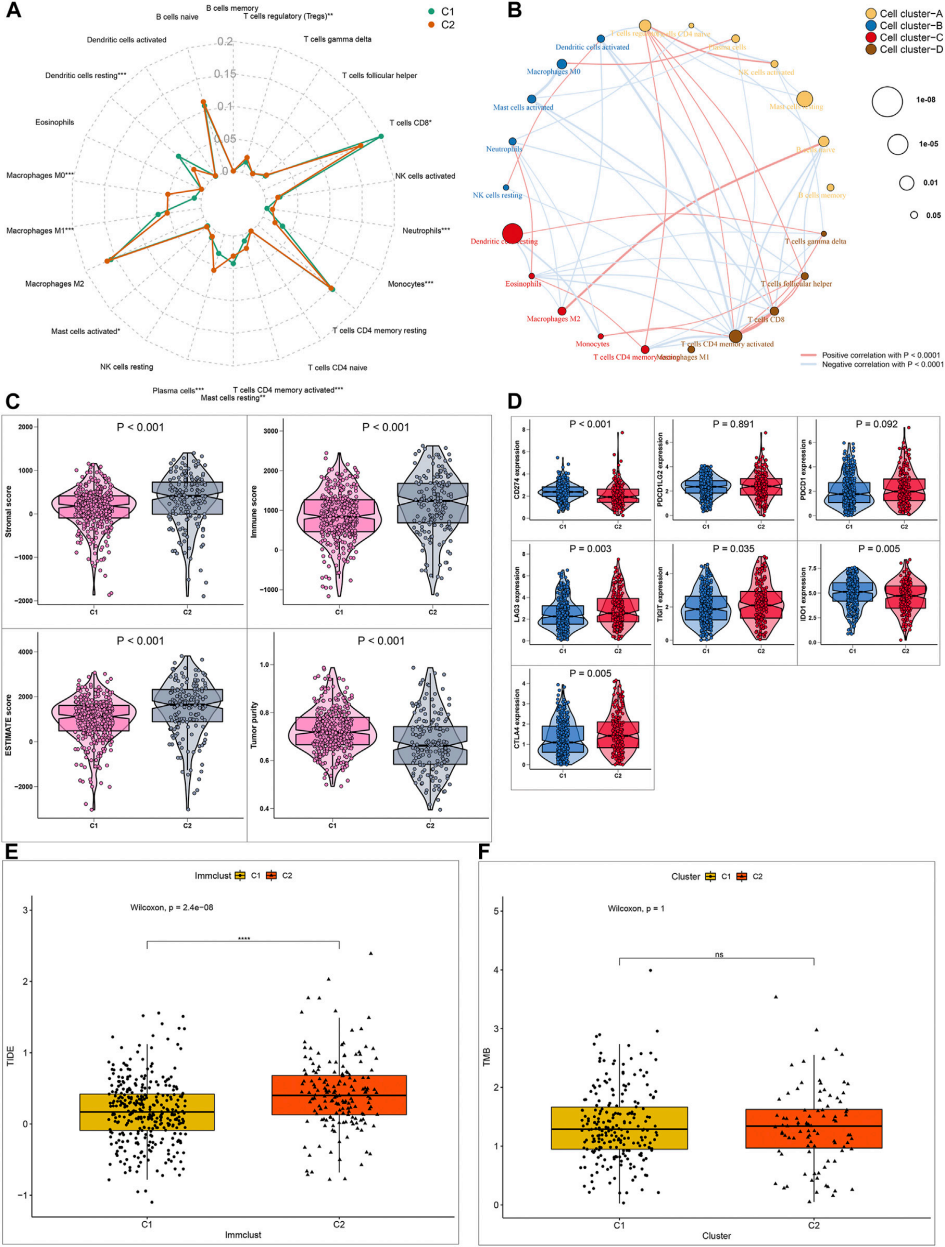
Differential sensitivity to immunotherapy between two ccRCC molecular subtypes **(A)** Radar map showing the differences in proportions of 22 immune cell types between C1 and C2 molecular subtypes **(B)** A network of the correlation between the levels of 22 kinds of tumor-infiltrating immune cells in ccRCC samples. The size of bubble is inversely proportional to *p*-value **(C)** Patterns of stromal cell scores, immune cell scores, ESTIMATE scores and tumor purity between subtypes **(D)** Expression levels of immune checkpoint markers in the two subtypes **(E, F)** Box plots showing the correlation between TIDE/TMB levels and molecular subtypes. *p < 0.05; **p < 0.01; ***p < 0.001; ****p < 0.0001; ns: no statistical significance.

Immunotherapy has been approved for the treatment of ccRCC. However, which group of patients responds to immunotherapy is still unknown. Here, we firstly assessed the differential sensitivity to immunotherapy between the two hypoxia-related molecular subtypes. The infiltration levels of 22 kinds of immune cells for each sample were detected utilizing the CIBERSORT algorithm. As a result, C2 displayed the higher infiltration levels of T cells regulatory (Tregs; *p* < 0.01), macrophages M0 (*p* < 0.001), mast cells activated (*p* < 0.05), plasma cells (*p* < 0.001), T cells CD4 memory activated (*p* < 0.001), neutrophils (*p* < 0.001) compared to C1 (Figure 3A). Meanwhile, C1 exhibited distinctly higher levels of dendritic cells resting (*p* < 0.001), macrophages M1 (*p* < 0.001), mast cells resting (*p* < 0.01), monocytes (*p* < 0.001), T cells CD8^+^ (*p* < 0.05) in comparison to C2. In Figure 3B, these immune cells were clustered into four cell clusters by hierarchical agglomerative clustering based on Euclidean distance and Ward’s linkage. There was a complex interaction network between different immune cells, indicating the complexity of tumor immune microenvironment. For example, the infiltration levels of macrophages M2 were positively correlated with B cells naïve in ccRCC tissues. The patterns of stromal scores, immune scores, ESTIMATE scores and tumor purity in each ccRCC sample were evaluated *via* ESTIMATE algorithm. The samples in C2 subtype had the relatively high levels of stroma, immune and ESTIMATE scores in comparison to C1 (all *p* < 0.001; Figure 3C). Furthermore, we investigated the lower levels of tumor purity in C2 than in C1 (*p* < 0.001). These suggested that C2 was more likely to experience a worse prognosis than C1. ICIs have been used for the first-line therapy of metastatic ccRCC (Shah et al., 2019). Nevertheless, not all patients may benefit from it. The patients’ response to immunotherapy was predicted by the TIDE algorithm. Higher expression levels of CD274 mRNA (*p* < 0.001) were found in C1 compared to C2 (Figure 3D). Meanwhile, LAG3 (*p* = 0.003), TIGIT (*p* = 0.035), IDO1 (*p* = 0.005) and CTLA4 (*p* = 0.05) mRNAs displayed higher expression levels in C2 than C1 (Figure 3D). Moreover, C2 displayed higher TIDE levels than C1 (Figure 3E; *p* = 2.4e-08). As such, our data showed that C2 was more likely to respond to immunotherapy compared to C1 (Figure 3E; *p* = 2.4e-08). High TMB usually indicates poor clinical outcomes and is a powerful predictor for immunotherapy response in ccRCC (Huang et al., 2020). However, there was no significant difference in TMB between subtypes (Figure 3F).

### Evaluation of the Expression Levels of CXC Chemokines in Two ccRCC Molecular Subtypes

**FIGURE 4:**
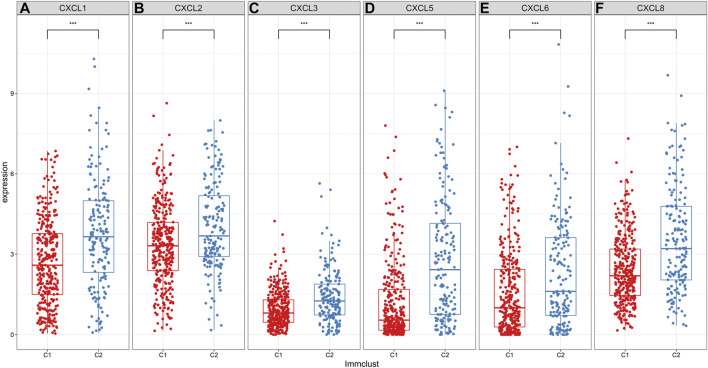
Differences in expression patterns of CXC chemokines between two ccRCC molecular subtypes. As depicted in the box plots, the expression levels of **(A)** CXCL1 **(B)** CXCL2 **(C)** CXCL3 **(D)** CXCL5 **(E)** CXCL6 and **(F)** CXCL8 are visualized in ccRCC samples between C1 and C2. ***p < 0.001.

Herein, we assessed the expression levels of CXC chemokines in ccRCC samples between C1 and C2 (Zeng et al., 2019). As a result, C2 exhibited the higher expression levels of CXCL1 (Figure 4A), CXCL2 (Figure 4B), CXCL3 (Figure 4C), CXCL5 (Figure 4D), CXCL6 (Figure 4E) and CXCL8 (Figure 4F) in comparison to C1 (all *p* < 0.001). Among them, a previous study has showed that low expression of CXCL1/2/3/5 was in relationship with a better prognosis for RCC patients, indicating that these chemokines could contribute to poor clinical outcomes for patients in C2 (Zeng et al., 2019).

### Differential Putative Chemotherapeutic Response Between Molecular Subtypes

**FIGURE 5:**
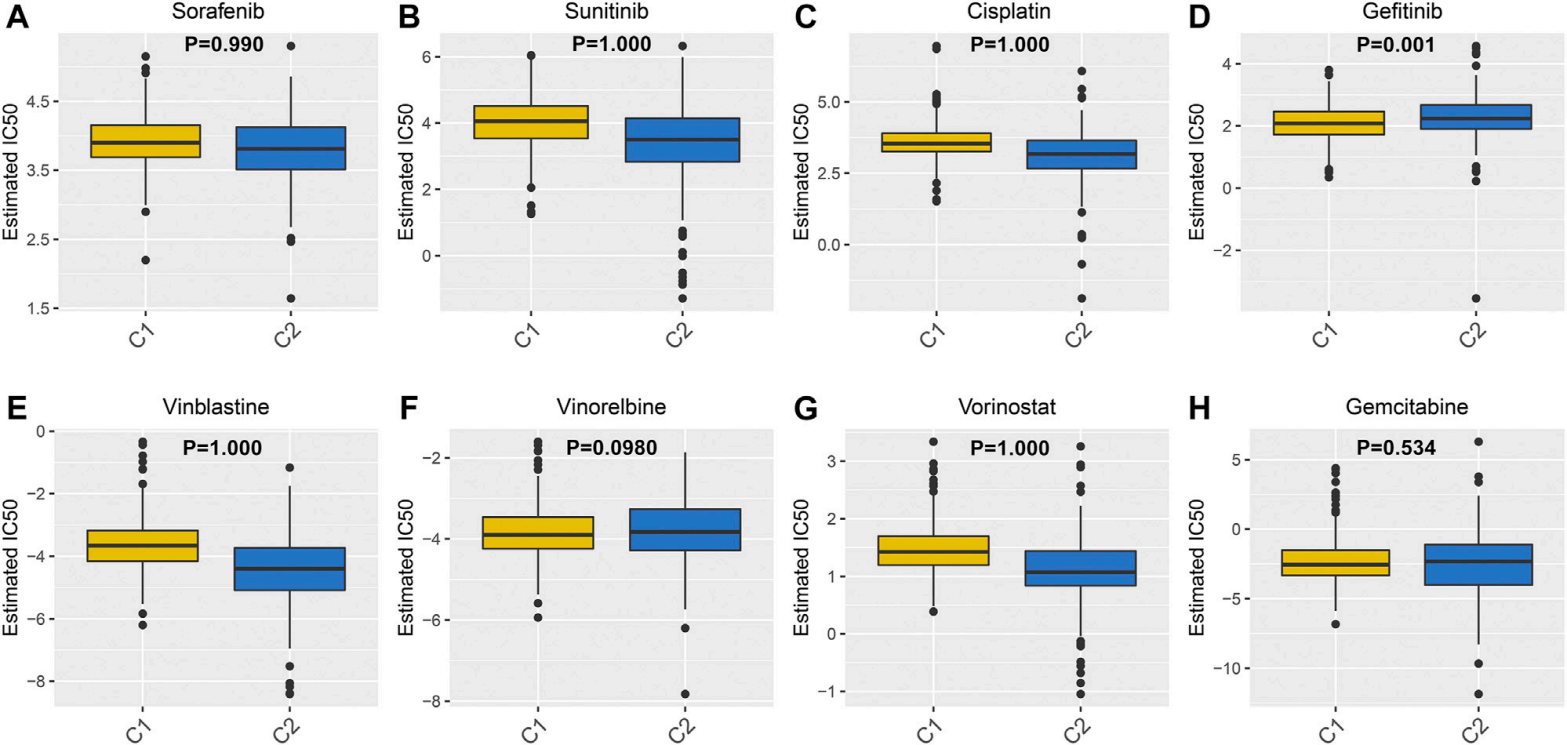
Differences in sensitivity to chemotherapy drugs between two ccRCC molecular subtypes. The box plots depicting the estimated IC50 values for **(A)** Sorafenib **(B)** sunitinib **(C)** Cisplatin **(D)** gefitinib **(E)** Vinblastine **(F)** Vinorelbine **(G)** Vorinostat and **(H)** Gemcitabine in ccRCC samples from the two molecular subtypes.

Drug resistance has become a major challenge in chemotherapy, involving various mechanisms. Hypoxia, as a key factor, affects cell expression programs and induces treatment resistance (Jing et al., 2019). Herein, GDSC database was employed to assess the differences in the sensitivity between the two hypoxia-related molecular subtypes to eight common chemotherapy drugs including Sorafenib (Figure 5A), sunitinib (Figure 5B), Cisplatin (Figure 5C), gefitinib (Figure 5D), Vinblastine (Figure 5E), Vinorelbine (Figure 5F), Vorinostat (Figure 5G) and Gemcitabine (Figure 5H). Drug response was defined based on IC50 values. The data suggested that C2 subtype was more sensitive to most of chemotherapy drugs such as Sorafenib, Sunitinib, Cisplatin, Vinblastine and Vorinostat compared to C1 subtype, indicating that patients in C2 subtype were more likely to benefit from above chemotherapy drugs. Meanwhile, C1 subtype had the higher sensitivity to Gefitinib, Gemcitabine and Vinorelbine than C2 subtype, indicating that patients in C1 subtype might respond to these chemotherapy drugs.

### CMap Analysis Identifies Candidate Inhibitors for ccRCC

**FIGURE 6:**
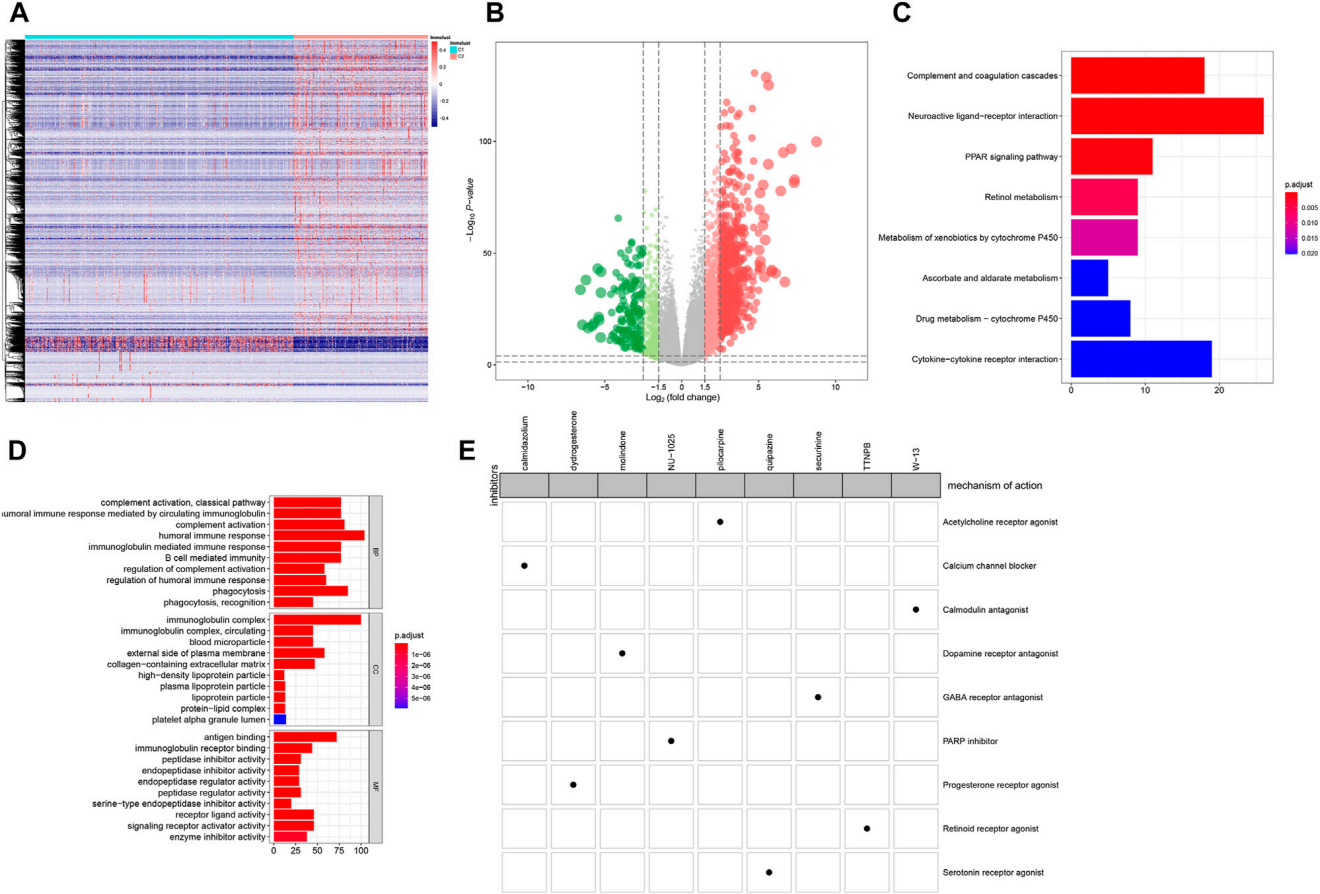
CMap analysis identifies candidate small molecular inhibitors for ccRCC **(A)** Heatmap depicting all DEGs between C1 (blue) and C2 (red) **(B)** Volcano plots up-regulated genes (red bubbles) and down-regulated genes (green bubbles) in C1 compared to C2 **(C)** Bar plots of the top ten enriched KEGG signaling pathways **(D)** Bar plots of the top ten GO function annotation analysis results including biological processes (BP), cellular component (CC) and molecular function (MF) categories. Red suggests high enrichment and blue suggests low enrichment **(E)** Heatmap demonstrating each inhibitor (perturbagen) and its shared mechanisms of action (rows) *via* the CMap database.

1,472 DEGs were identified between C1 and C2 (Figures 6A,B). Among them, there were 1,203 up- and 269 down-regulated genes in C2 compared to C1 (Supplementary Table 4). As shown in KEGG enrichment analysis results, ccRCC-related signaling pathways such as complement and coagulation cascades, neuroactive ligand-receptor interaction, cytokine-cytokine receptor interaction and PPAR signaling pathway were significantly enriched by these DEGs (Figure 6C). GO function annotation analysis revealed that these DEGs could possess immune-related functions (Figure 6D). Through the CMap, we screened out nine small molecule inhibitors (pilocarpine, quipazine, calmidazolium, dydrogesterone, securinine, molindone, W-13, TTNPB and NU-1025). Among them, pilocarpine (enrichment = −0.865 and *p* = 0.00062) and quipazine (enrichment = −0.704 and *p* = 0.01578) could become candidate small molecule drugs for all ccRCC patients (Figure 6E). According to MoA analysis, nine mechanisms of actions (acetylcholine receptor agonist, serotonin receptor agonist, calcium channel blocker, progesterone receptor agonist, GABA receptor antagonist, dopamine receptor antagonist, calmodulin antagonist, retinoid receptor agonist and PARP inhibitor) shared the above small molecule inhibitors, indicating that nine small molecule inhibitors might suppress ccRCC progression through mediating these mechanisms of actions.

### Development of a Subtype-specific Prognostic Five-Gene Signature for ccRCC

**FIGURE 7:**
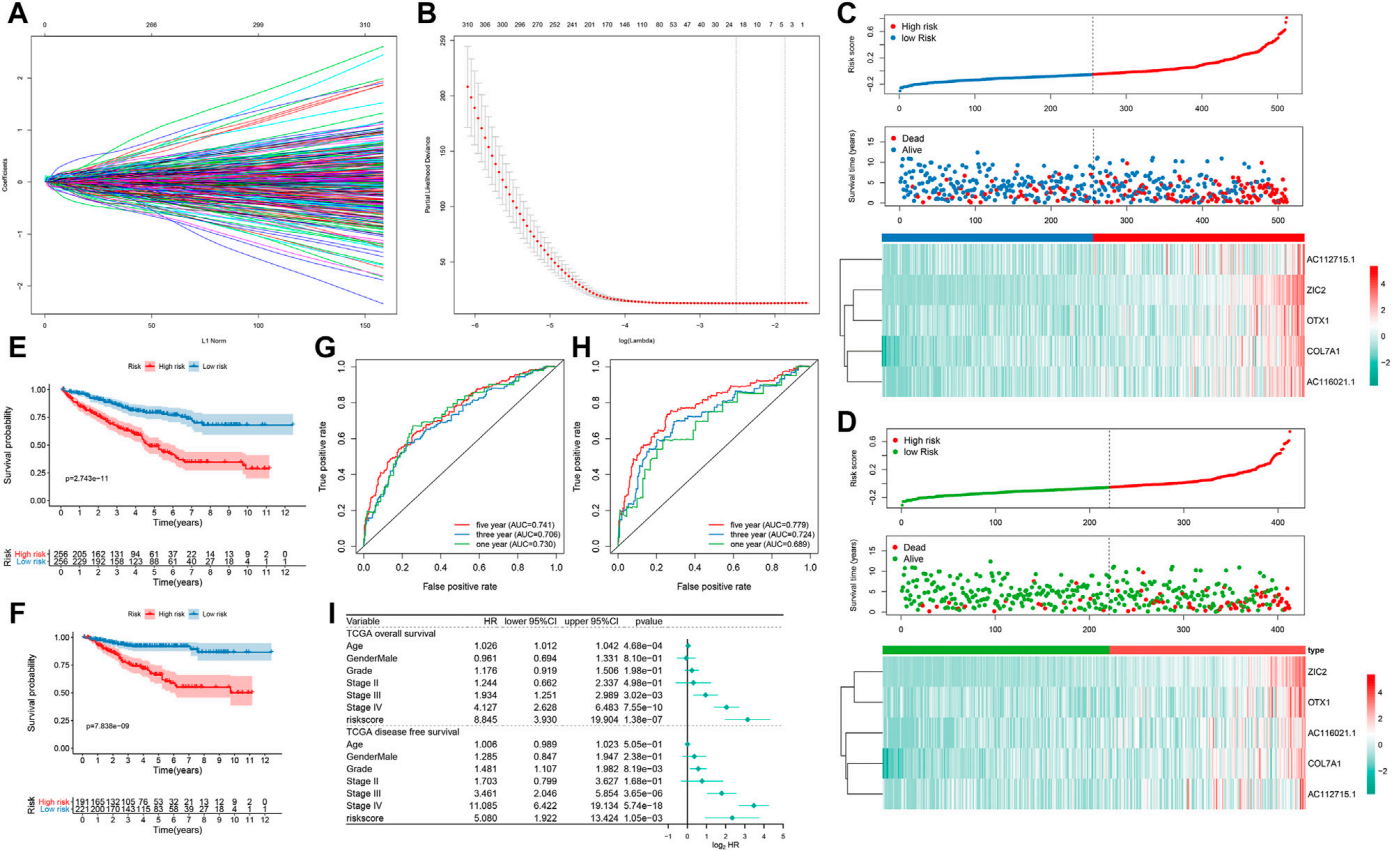
Development of a prognostic five-gene signature for ccRCC in TCGA dataset **(A)** 20-time cross-validation for tuning parameter selection in the LASSO Cox model **(B)** Plots of the LASSO coefficients **(C)** The risk score rank (up), distribution of survival status (alive or dead; middle) and expression patterns of five genes in high- and low-risk groups **(D)** The risk score rank (up), distribution of survival status (diseased or disease-free; middle) and expression patterns of five genes (down) in high- and low-risk groups **(E, F)** Kaplan-Meier OS and DFS curve for high- and low-risk groups **(G)** Time-dependent ROC curves for one-, three- and five-years OS time **(H)** Time-dependent ROC curves for one-, three- and five-years DFS time **(I)** Forest plots showing the multivariate Cox regression analyses results of the risk score and clinical factors with OS and DFS.

Totally, 902 prognosis-related DEGs between subtypes were identified for ccRCC. Under LASSO Cox regression analysis (Figures 7A,B), a five-gene signature was constructed, composed of COL7A1, ZIC2, AC116021.1, AC112715.1 and OTX1. The risk score of each sample was calculated and all ccRCC patients were separated into high- and low groups in accordance with the cutoff values of risk scores. The higher the risk score, the greater the number of patients with dead (Figure 7C) or disease status (Figure 7D). High-risk group had the higher expression levels of these genes than low-risk group (Figures 7C,D). Patients with high-risk score exhibited a poorer OS (*p* = 2.743e-11; Figure 7E) and DFS (*p* = 7.838e-09; Figure 7F). As shown in ROC curves, AUCs for one-, three- and five-years OS were 0.730, 0.706, and 0.741, suggesting the well performance of the risk score for prediction of OS (Figure 7G). Also, AUCs for one-, three- and five-years DFS were 0.689, 0.724, and 0.779, confirming its predictive efficacy for DFS (Figure 7H). Our multivariate cox regression analysis demonstrated that this signature could independently predict ccRCC patients’ OS and DFS (Figure 7I).

### A Nomogram Integrating Subtype-specific Signature and Clinical Factors Improves Predictive Power for ccRCC Prognosis

**FIGURE 8:**
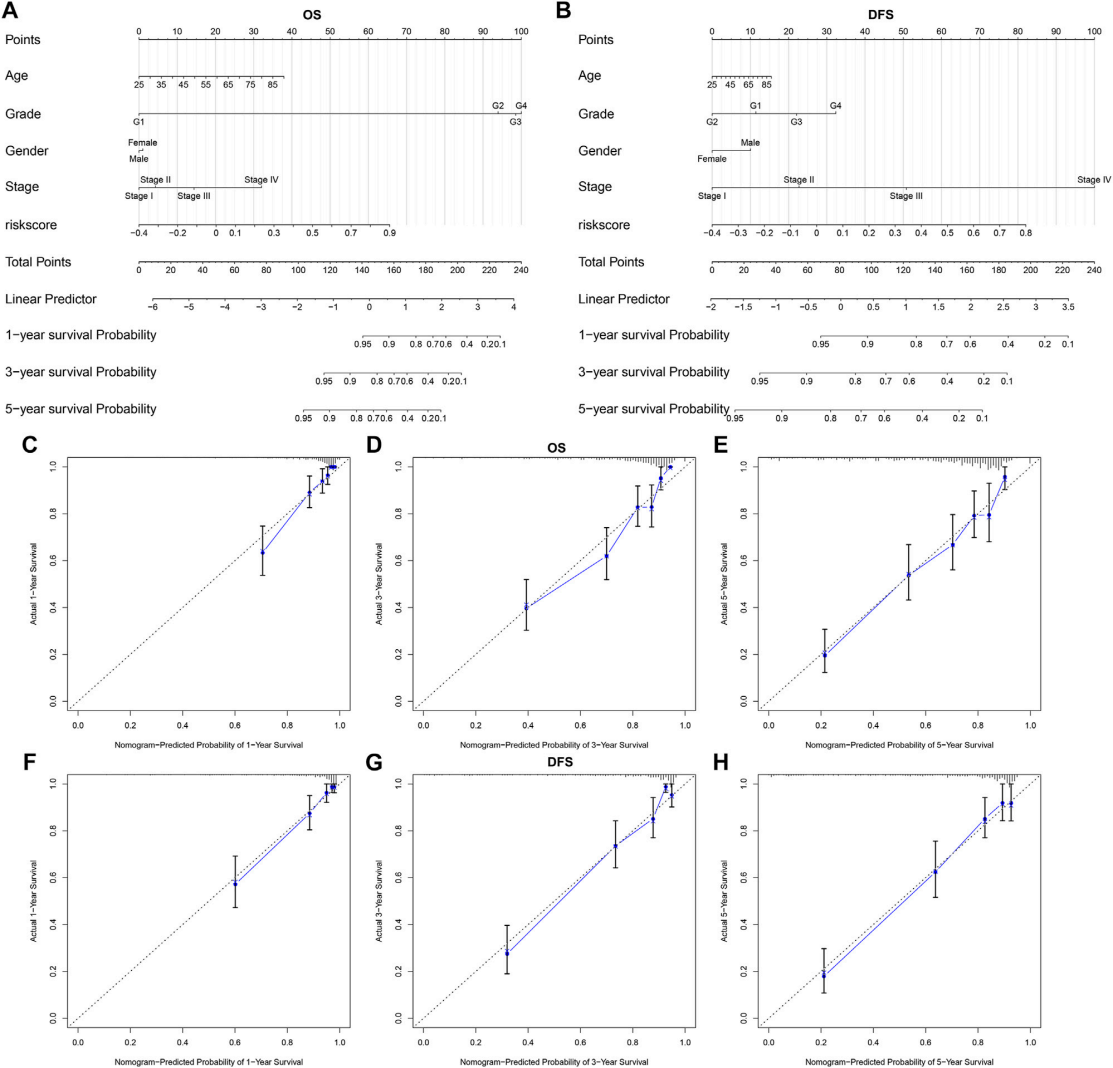
A nomogram incorporating subtype-specific signature and clinical factors improves predictive efficacy for ccRCC prognosis **(A, B)** Construction of a nomogram combining the subtype-specific signature and clinical features for prediction of OS and DFS. Calibration plots displayed the actual and nomogram-predicted probability of one-, three- and five-years OS **(C–E)** and DFS **(F–H)**.

We constructed a nomogram by combining the five-gene signature and clinical factors including age, grade, gender, and stage for predicting ccRCC patients’ OS (Figure 8A) and DFS (Figure 8B). We further evaluated whether the integration of the five-gene signature and clinical factors could boost the predictive efficiency for ccRCC prognosis in TCGA dataset. Calibration plots confirmed that the nomogram-predicted probabilities of one- (Figure 8C), three- (Figure 8D) and five-years (Figure 8E) OS had high consistency with the actual survival. Moreover, the nomogram-predicted probabilities of one- (Figure 8F), three- (Figure 8G) and five-years (Figure 8H) DFS was close to the actual survival. Collectively, the nomogram integrating the five-gene signature, age, grade, gender, and stage could enhance the predictive power of ccRCC patients’ prognosis.

## Discussion

Emerging first-line treatment options such as targeted drugs and immunotherapy have significantly improved the prognosis of ccRCC patients with high risk, for whom chemoradiotherapy has shown limited efficacy (Atkins and Tannir, 2018). It has been widely recognized the differences in response to therapy due to the molecular and histologic heterogeneity of ccRCC (Luo et al., 2019). In this study, we characterized two hypoxia-related molecular subtypes for ccRCC with distinct clinical outcomes and response to immunotherapy and targeted therapy based on multi-omics analysis.

Hypoxia is a key feature of the tumor microenvironment, driving tumor aggressiveness (Balamurugan, 2016). To adapt to hypoxia, the expression of hypoxia-related genes changes accordingly. Based on prognosis-related hypoxia genes, we characterized two molecular subtypes with distinct molecular subtypes in TCGA and ICGC databases. The hypoxia-related classifier may become a practical and reliable predictive tool, which could complement the current staging system for predicting ccRCC prognosis. The differences in survival status, stage, gender, and grade did not reduce the accuracy of the classifier in predicting patients’ prognosis. Specifically, it has been acknowledged that male patients exhibit more aggressive characteristics as well as poorer OS compared to females (Brannon et al., 2012). Differential somatic mutations and CNVs were detected between subtypes. Consistent with previous study, VHL (50%) and PBRM1 (43%) mutations commonly occur in ccRCC (Carril-Ajuria et al., 2019). The loss of VHL tumor suppressor gene is the most common genetic feature of ccRCC, which improves the expression of target genes of hypoxia-inducible factors (HIFs), thereby affecting metabolism and signal transduction for ccRCC cells (Zhang et al., 2018).

Despite various gene mutations gain the incidence of ccRCC, the tumor microenvironment has a critical influence on tumor development and immune response. In the tumor immune microenvironment, there were distinct differences immune cell infiltrations between subtypes. Tumor-infiltrating immune cells are linked to clinical outcomes as well as response to immunotherapy. Our characterized subtypes were associated with immune infiltration patterns in ccRCC. Particularly, ccRCC is the tumor type with the highest infiltration levels of T cells (Şenbabaoğlu et al., 2016). There were distinct differences in the infiltration of T cell subpopulations between subtypes. Immune status affects ccRCC patients’ clinical outcomes. Functional enrichment analysis revealed that DEGs between subtypes could be involved in immune response. Compared to C1, C2 had higher stromal/immune scores and lower tumor purity. Furthermore, high stromal/immune scores and low tumor purity of ccRCC patients have been found to be significantly associated with poor prognosis (Xu et al., 2019). Targeted therapeutics, such as VEGF receptors and mTOR inhibitors, can distinctly prolong the survival time of metastatic ccRCC patients (Xu et al., 2019). Nevertheless, most of patients do not have targetable mutations. Immune checkpoint targets provide another promising treatment strategy. However, the hypoxic microenvironment of tumors can reduce immune activity. Here, we found that C2 subtype had higher levels of ICIs than C1. For example, LAG3+ T cells is a sign of T cell exhaustion that is a key factor for immunosuppressive properties and is associated with advanced ccRCC (Wang et al., 2019). Based on the TIDE algorithm, it was estimated that C2 possessed higher potential response of immune-checkpoint blockade (ICB) therapy. Thus, ICB therapy may be efficacious for C2 subtype of ccRCC patients.

Prognostic biomarkers related to the tumor immune microenvironment may provide promising prospects for identifying novel molecular targets and improving patients’ clinical outcomes undergoing immunotherapy (Burger and Kipps, 2006). DEGs between subtypes were significantly enriched in chemokine-chemokine receptor interaction. Low expression of CXCL1/2/3/5 chemokines exhibits better clinical outcomes in RCC (Zeng et al., 2019). Our data showed that the expression of these chemokines in subtype C1 was significantly lower than that of subtype C2, indicating that chemokines could promote tumor escape of ccRCC of C2 subtype, thereby leading to poorer prognosis.

Treatment based on individual tumor characteristics provides the possibility to improve the different clinical outcomes of patients due to tumor heterogeneity in ccRCC (Hu et al., 2020). Changes in the cancer genome in response to hypoxia markedly affect the response to anticancer therapies (Ye et al., 2019). It is reasonable to predict the treatment response to chemotherapy, which can reduce the cost of treatment and improve the prognosis of patients. This study demonstrated that C2 subtype exhibited higher sensitivity to most of chemotherapeutic drugs being used [such as sorafenib and sunitinib have been approved for treating metastatic RCC (Hsieh et al., 2017)] or developed than C1 subtype, indicating that patients in C2 could be more suitable for above therapies, which can provide an available strategy to select patients who benefit from a particular therapy.

Signatures based on gene expression have not yet been incorporated into routine clinical practice for ccRCC. Compared with the traditional method using gene expression levels, LASSO algorithm eliminates the requirement for data preprocessing, which has been proven to produce reliable results including cancer classification (Li et al., 2017). This study constructed a subtype-specific signature using LASSO Cox regression analysis. Following validation, this signature could robustly and independently predict OS and DFS of ccRCC patients. Furthermore, we constructed the nomogram combining the signature and other clinical factors. The prediction system can guide the establishment of personalized examination procedures for ccRCC patients and boost the effective use of medical resources.

However, this study is based on the retrospective design. This classifier system will be verified in our future multi-center prospective research. In conclusion, the two hypoxia-related molecular subtypes as well as subtype-specific nomogram could be utilized to guide the current clinical application to maximize patients’ benefit from immunotherapy or targeted therapy.

## Conclusion

Collectively, we constructed two hypoxia-related molecular subtypes with distinct prognosis for ccRCC, which could assist manage risk assessment and provide valuable insights for the immunotherapy and targeted therapy strategies of ccRCC.

## Data Availability

Publicly available datasets were analyzed in this study. This data can be found here: TCGA, http://cancergenome.nih.gov/&lt;/b&gt.

## Glossary

ccRCC: clear cell renal cell carcinoma
CI: confidence interval
CMap: Connectivity map
CNV: copy number variation
DEGs: differentially expressed genes
DFS: disease-free survival
ESTIMATE: Estimation of stromal and immune cells in malignant tumors using expression data
FC: fold change
FDR: false discovery rate
GDSC: Genomics of Drug Sensitivity in Cancer
GISTIC: Genomic Identification of Significant Targets in Cancer
GO: Gene Ontology
GSEA: Gene Set Enrichment Analysis
GSVA: Gene set variation analysis
HR: hazard ratio
IBERSORT: Cell type identification by estimating relative subsets of RNA transcripts
IC50: the half maximal inhibitory concentration
ICGC: International Cancer Genome Consortium
ICI: immune checkpoint inhibitors
KEGG: Kyoto Encyclopedia of Genes and Genomes
LASSO: least absolute shrinkage and selection operation
limma: linear models for microarray data
MoA: CMap mode-of-action
MSigDB: Molecular Signatures database
NMF: non-negative matrix factorization
OS: overall survival
PCA: principal components analysis
RCC: renal cell carcinoma
RNA-seq: RNA sequencing
ROCs: receiver operating characteristic curves
TCGA: The Cancer Genome Atlas
TIDE: Tumor Immune Dysfunction and Exclusion
TMB: tumor mutation burden
t-SNE: t-distributed stochastic neighbor embedding.
