# Supplementary figures and images for: Characterization of Hypoxia-Related Molecular Subtypes in Clear Cell Renal Cell Carcinoma to Aid Immunotherapy and Targeted Therapy via Multi-Omics Analysis
Source: Front Mol Biosci. 2021 Jun 25;8:684050. doi: 10.3389/fmolb.2021.684050 (PMC8267011; doi:10.3389/fmolb.2021.684050)

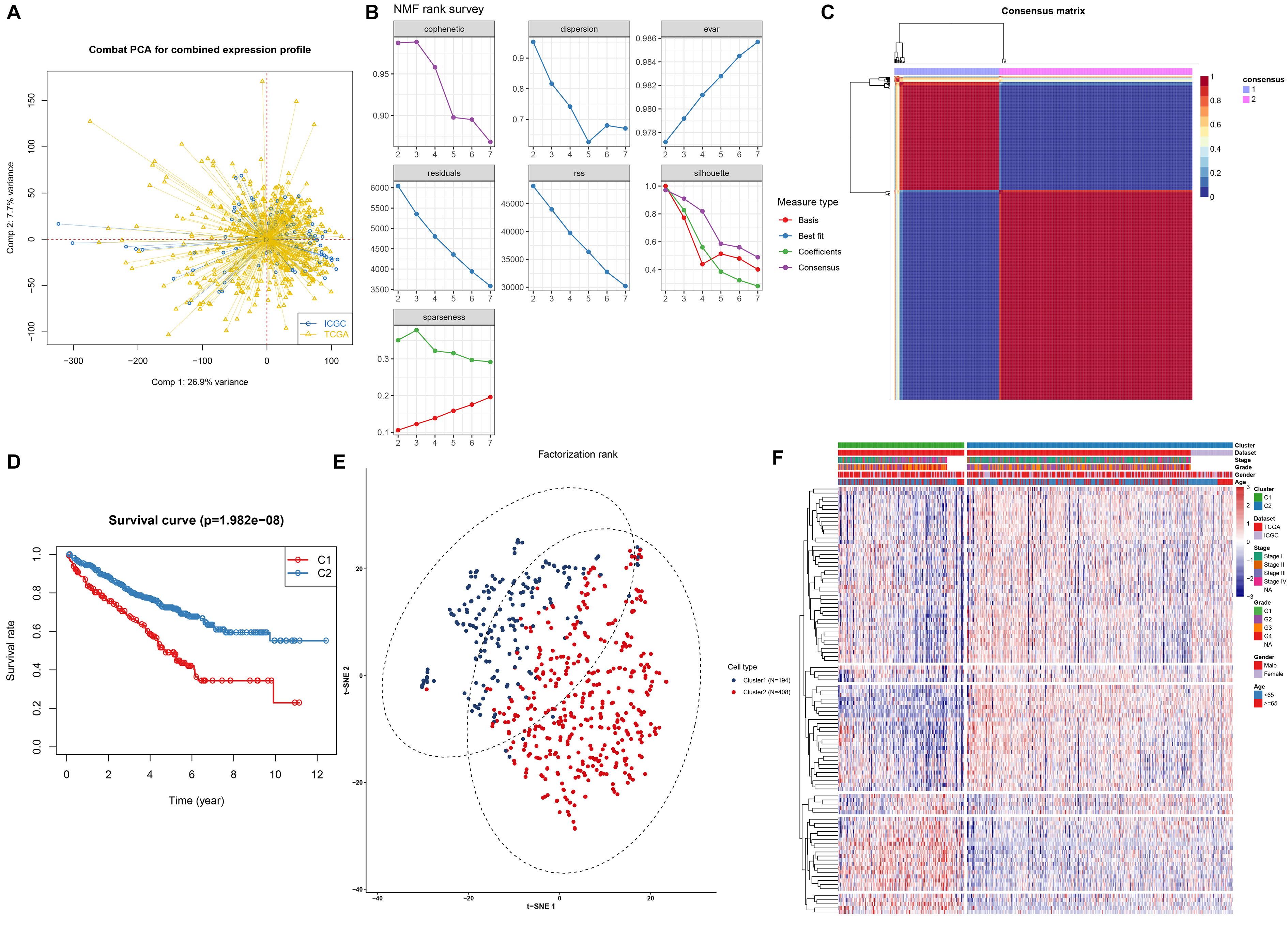

Supplement: Supplementary file 3 [file Image2.TIF]

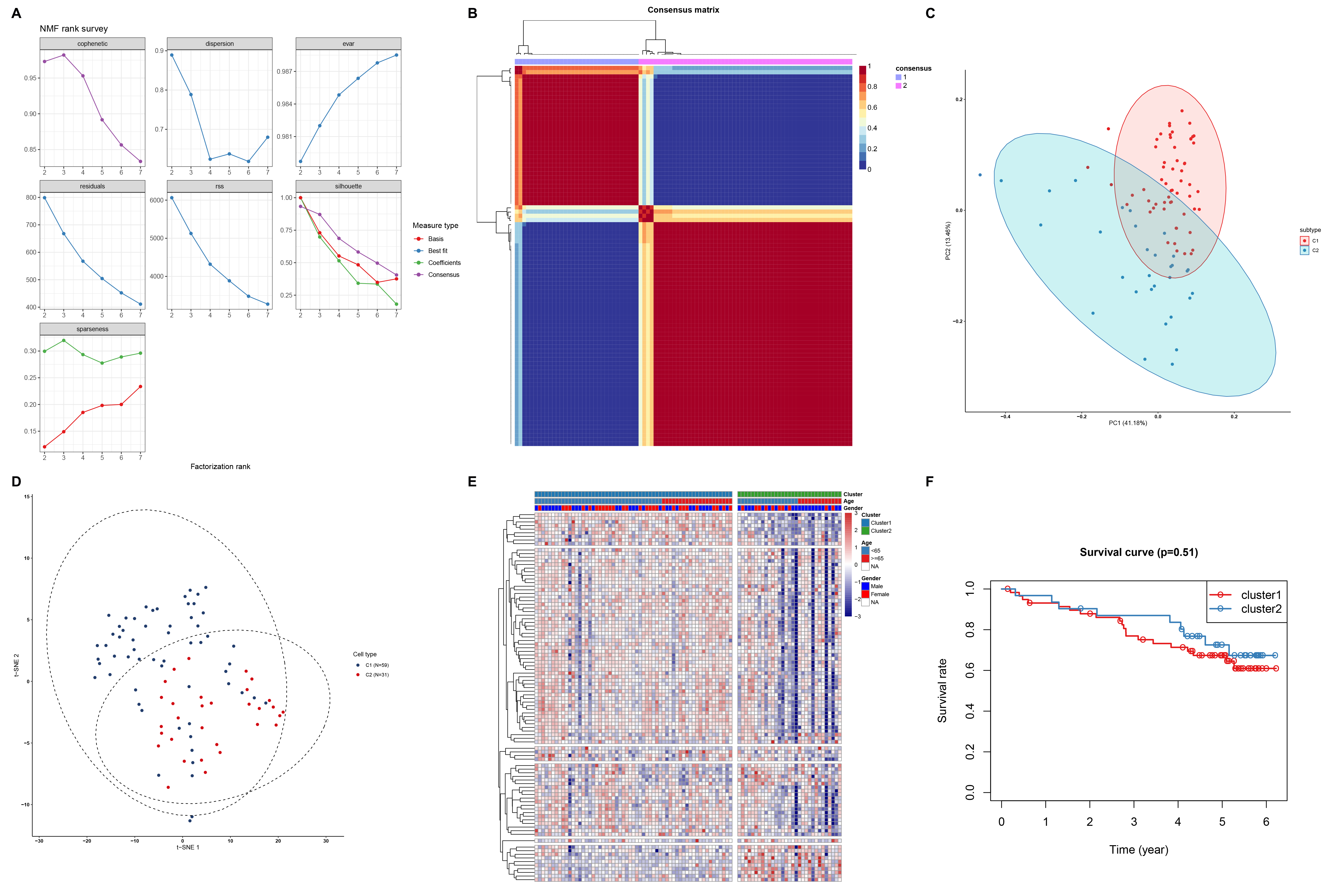

Supplement: Supplementary file 4 [file Image1.TIF]
